# Comparison of Chemical Sensitivity of Fresh and Long-Stored Heat Resistant *Neosartorya fischeri* Environmental Isolates Using BIOLOG Phenotype MicroArray System

**DOI:** 10.1371/journal.pone.0147605

**Published:** 2016-01-27

**Authors:** Jacek Panek, Magdalena Frąc, Nina Bilińska-Wielgus

**Affiliations:** Institute of Agrophysics, Polish Academy of Sciences, Department of Plant and Soil System, Laboratory of Molecular and Environmental Microbiology, Doświadczalna 4, 20–290 Lublin, Poland; Agricultural University of Athens, GREECE

## Abstract

Spoilage of heat processed food and beverage by heat resistant fungi (HRF) is a major problem for food industry in many countries. *Neosartorya fischeri* is the leading source of spoilage in thermally processed products. Its resistance to heat processing and toxigenicity makes studies about *Neosartorya fischeri* metabolism and chemical sensitivity essential. In this study chemical sensitivity of two environmental *Neosartorya fischeri* isolates were compared. One was isolated from canned apples in 1923 (DSM3700), the other from thermal processed strawberry product in 2012 (KC179765), used as long-stored and fresh isolate, respectively. The study was conducted using Biolog Phenotype MicroArray platforms of chemical sensitivity panel and traditional hole-plate method. The study allowed for obtaining data about *Neosartorya fischeri* growth inhibitors. The fresh isolate appeared to be much more resistant to chemical agents than the long-stored isolate. Based on phenotype microarray assay nitrogen compounds, toxic cations and membrane function compounds were the most effective in growth inhibition of *N*. *fischeri* isolates. According to the study zaragozic acid A, thallium(I) acetate and sodium selenate were potent and promising *N*. *fischeri* oriented fungicides which was confirmed by both chemical sensitivity microplates panel and traditional hole-plate methods.

## Introduction

*Neosartorya fischeri* is a heat resistant fungus (HRF) and is the leading cause of spoilage in thermally processed food and beverage, especially fruit products in many countries [1]. First identification of *Neosartorya fischeri* from canned strawberries took place in 1963 [2]. Certain strains of *N*. *fischeri* have been reported as able to produce mycotoxins, such as verruculogen, tryptoquivaline and fumitremorgins A, B and C [3]. *N*. *fischeri* is able to survive at least 75°C for more than 30 minutes, that is the way these fungi are included to heat resistant organisms [1,4–8]. Ascospores of *N*. *fischeri* may spoil heat processed products by their germination and growth, even under microaerobic conditions [7]. The toxigenicity of these moulds constitutes hazard to public health [3,4,9]. Contamination of agricultural raw materials is often a result of their contact with the soil.

Finding a way to decrease the risk of HRF food spoilage should be a major concern. One of possible ways to prevent food from HRF contamination and, in consequence, spoilage is finding substances that can effectively stop the growth of these fungi.

Morphological and occurrence analysis of *N*. *fischeri* has provided useful information about this pathogen, however studies concerning chemical resistance and sensitivity of *N*. *fischeri* are a novelty. There are also no studies concerning the comparison of chemical sensitivity profiles between long-stored and fresh-isolated strains. This study is the first attempt to determine the chemical sensitivity of *N*. *fischeri* against antibiotics, chemicals and osmolytes using Phenotype Microarray. With the development of phenotype microarrays (PM), the high-throughput determination of fungal chemical phenotype is now possible [4,10]. Biolog PM is a complex platform to facilitate the meta-analysis of phenomics data of microbial organisms [11]. The complete PM panel for fungal cells contains fifteen pre-formulated 96-well plates (PM1 to PM10 and PM21 to PM25) pre-coated with chemical compounds or combinations thereof. PM plates may be used in studies on utilization of various sources of carbon (PM1-2), nitrogen (PM3, PM6-PM8) and phosphorus or sulphur (PM4). They can also be used to determine the sensitivity to stresses, such as ions or osmolytes stress (PM9), pH (PM10), and chemical agents (PMs 21–25). PM platform is able to monitor cellular metabolism with the use of colorimetric reporter system. Metabolic measurement is based on direct reading of tetrazolium-based dyes reduction to formazan in each well [12,13]. However, the traditional hole-plate methods should be used as reference assay, useful in verifying the results and strengthen the impact of chemicals on fungi.

The aim of this study was the comparison of the chemical sensitivity of two *N*. *fischeri* isolates, using Biolog PM chemical sensitivity panel (PMs 21–25). The results of PM assay were verified with traditional hole-plate zone of inhibition agar diffusion method. In this study we used a long-stored *N*. *fischeri* (DSM3700) from international collection and fresh, newly isolated *N*. *fischeri* (KC179765). It is known that fungi may lose their ability to break down different substrates due to key gene loss. It is tempting to suggest that fungi may also have tendency to lose or gain resistance to different chemicals during storage due to changes in genetic or metabolic pattern. We assume that study of fresh isolates that may be actually found in heat-treated products ensures that real threat is studied. The significant impact of heat resistant fungi on food industry makes studies on their chemical sensitivity extremely urgent.

## Materials and Methods

### 2.1. Fungal isolates

*N*. *fischeri* DSM3700 was obtained from the Leibniz Institute DSMZ-German Collection of Microorganisms and Cell Cultures (DSM3700). DSM3700 is an isolate originally isolated from canned apples by C. Wehmer in 1923, deposited and identified as *N*. *fischeri* by K.B. Raper 1965 [14]. In presented study we use *N*. *fischeri* DSM3700 as long-stored isolate. *N*. *fischeri* KC179765 was isolated from heat processed strawberry product in 2012 and we use it as fresh isolate. The isolate was identified at species level as *N*. *fischeri* based on the amplification and sequencing of the D2 region of the nuclear large-subunit (LSU) ribosomal RNA gene, using MicroSeq™ D2 rDNA Fungal Identification System (ABI). The sequence was deposited in GenBank of National Center for Biotechnology Information (NCBI; http://www.ncbi.nlm.nih.gov) under the KC179765 accession number [15].

### 2.2. Experimental procedures

The chemical sensitivity profile of studied *N*. *fischeri* isolates was measured using Biolog Phenotype MicroArrays chemical sensitivity panel PM21-PM25. Chemical sensitivity panel contains five PM (PMs 21–25) plates each of 96 wells. Each plate contains 24 different chemical agents (for a complete content description of each well in PM21-25 plates, see S1 Table) in 4 different concentrations each. The inhibitory chemicals in PM plates are provided in a titrated series of 4 wells, arranged with increasing concentrations going from left to right. To evaluate the resistance of isolates not only to individual compounds but also to their group we divided studied chemicals from whole panel (PMs 21–25) in 8 groups based on their structure and function: anions, cations, cyclic compounds, organic compounds, membrane function compounds, chelators, antibiotics and nitrogen compounds (S2 Table).

Cell suspensions were prepared from *N*. *fischeri* DSM3700 and *N*. *fischeri* KC179765 colonies cultured on malt extract agar plates for several days at 26°C, until good growth and ascospores were obtained. Then ascospores were removed from the surface of agar plates using a sterile swab by gently rubbing across the surface and transferred into sterile inoculating fluid (FF-IF, Biolog^TM^). Turbidity of suspension was measured and ascospores were added to obtain uniform suspension until it reached density of 62% transmittance, according to the manufacturer protocol. 300μl of ascospores suspension was transferred to 143.7 ml PM inoculation fluid containing FF-IF fluid, 0.67 grams of yeast nitrogen base and 12.81 grams of D-glucose. Then 100 μl of fungal inoculum was transferred into each well of particular PM plates. Inoculated PM plates were incubated in OmniLog incubator (Biolog^TM^) at 26°C for 96 hours. Data were automatically collected every 15 minutes.

To verify the results of chemical sensitivity performed by Biolog Phenotype MicroArrays a hole-plate zone of inhibition analysis was conducted. The following substances presented inhibitory effect on both tested *N*. *fischeri* isolates: zaragozic acid A, thallium(I) acetate, 3-amino-1,2,4-triazole, hydroxyurea, thiourea, copper(II) sulfate, sodium fluoride and sodium selenate were tested using hole-plate method. The *N*. *fischeri* DSM3700 and *N*. *fischeri* KC179765 isolates were inoculated on 90 mm Petri dishes with potato dextrose agar (PDA) medium. Then, 8 mm diameter holes were cut in the middle of each PDA medium in each plate. After that, 100μl of studied substances in 4 concentrations each: 1 mg/ml, 0.1 mg/ml, 0.01 mg/ml and 0.001mg/ml were added to each hole. The study were conducted in three replications. The results were collected after 24, 48 and 72 hours as a diameter of growth inhibition zone.

### 2.3. Statistical analysis

The data were processed using PM kinetic and parametric analysis v1.3 software and OPM library, and visualized with OPM level plot function [16,17]. The experiments were carried out in two biological replications for each isolate. Dataset included 9600 readings for both isolates. The well color development was measured 5 times every 15 minutes after 72 hours of incubation. The average values of color development from all readings for each chemicals were used for data analysis. We established thresholds for positive response of fungal growth at 60 Omnilog units. Analysis of variance (ANOVA) was used to determine the differences in inhibiting effect of individual chemical agents and their groups on the fungal isolates. Post-hoc analyses were performed using a Tukey test (HSD) analysis. All data were presented as 95% confidence intervals. Statistical significance was established at P < 0.05. Statistical analysis were performed using Statistica software (version 10.0).

## Results

**Fig 1:**
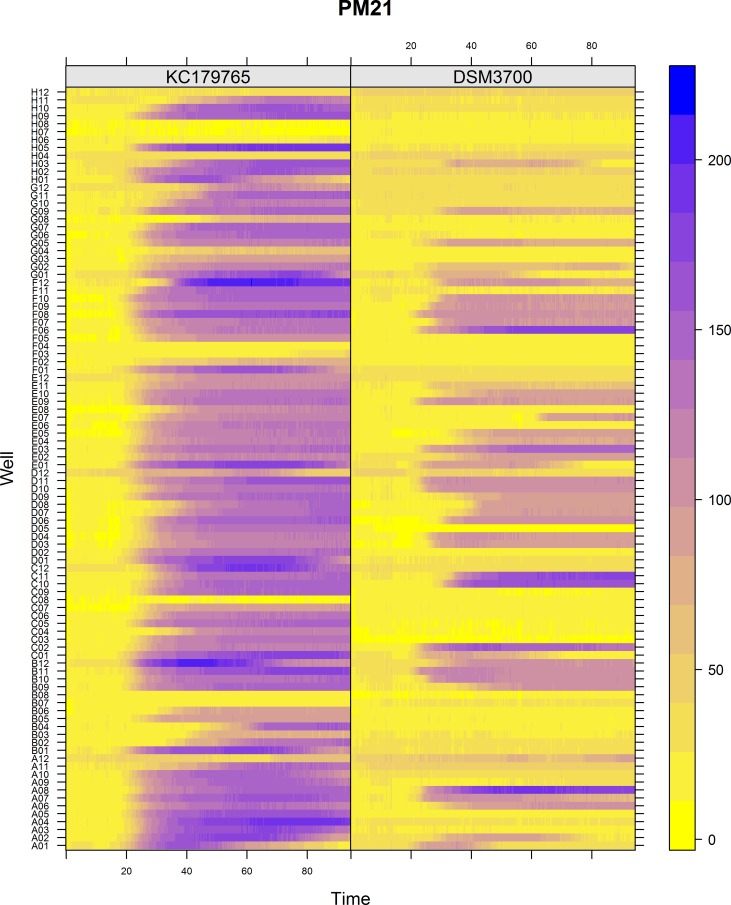
Visualization of *N*. *fischeri* DSM3700 and *N*. *fischeri* KC179765 growth kinetics on plate PM21. The growth values are represented by a color range as given in the scale bar on the right of the figure. The growth value increases from the yellow to the blue color. Yellow color represents growth inhibition.

**Fig 2:**
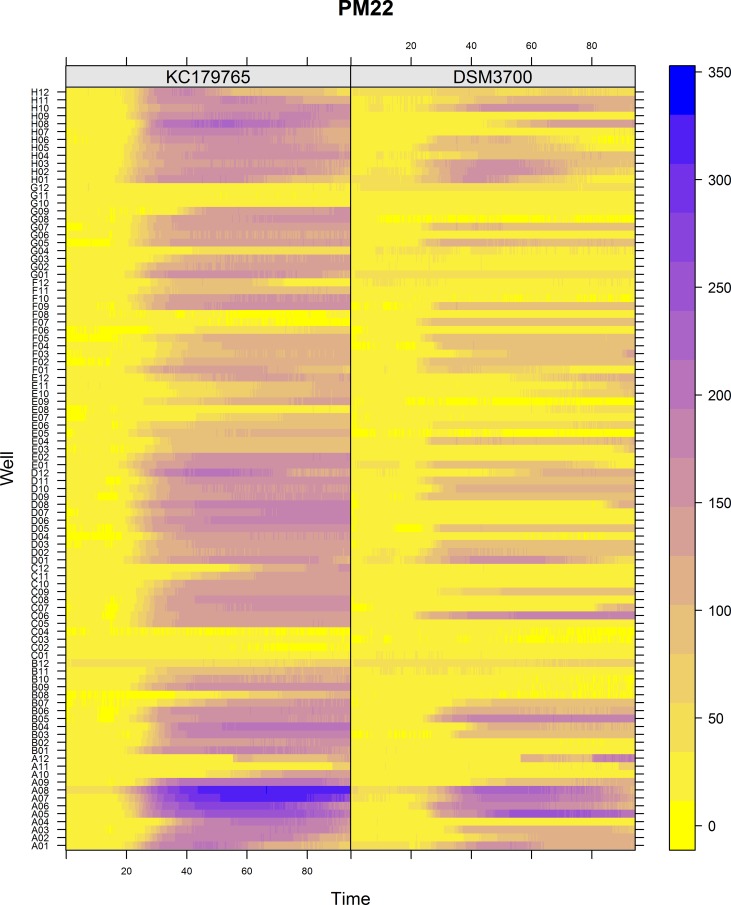
Visualization of *N*. *fischeri* DSM3700 and *N*. *fischeri* KC179765 growth kinetics on plate PM22. The growth values are represented by a color range as given in the scale bar on the right of the figure. The growth value increases from the yellow to the blue color. Yellow color represents growth inhibition.

**Fig 3:**
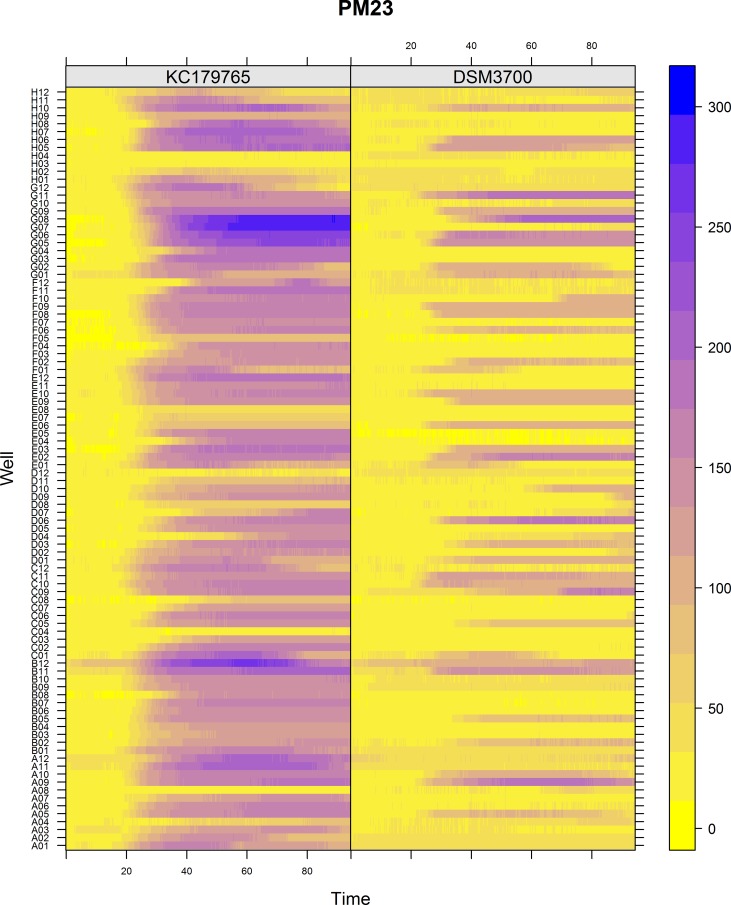
Visualization of *N*. *fischeri* DSM3700 and *N*. *fischeri* KC179765 growth kinetics on plate PM23. The growth values are represented by a color range as given in the scale bar on the right of the figure. The growth value increases from the yellow to the blue color. Yellow color represents growth inhibition.

**Fig 4:**
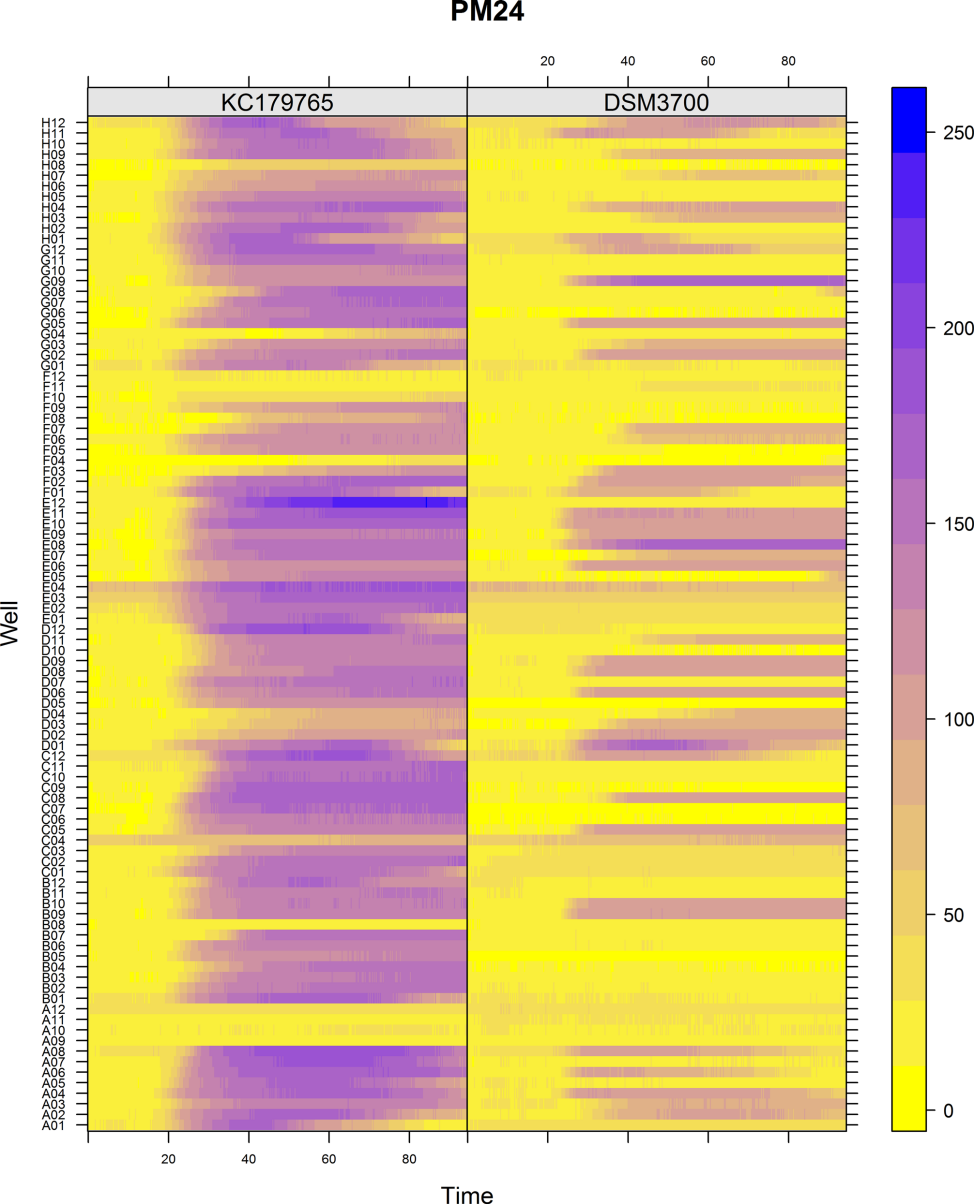
Visualization of *N*. *fischeri* DSM3700 and *N*. *fischeri* KC179765 growth kinetics on plate PM24. The growth values are represented by a color range as given in the scale bar on the right of the figure. The growth value increases from the yellow to the blue color. Yellow color represents growth inhibition.

**Fig 5:**
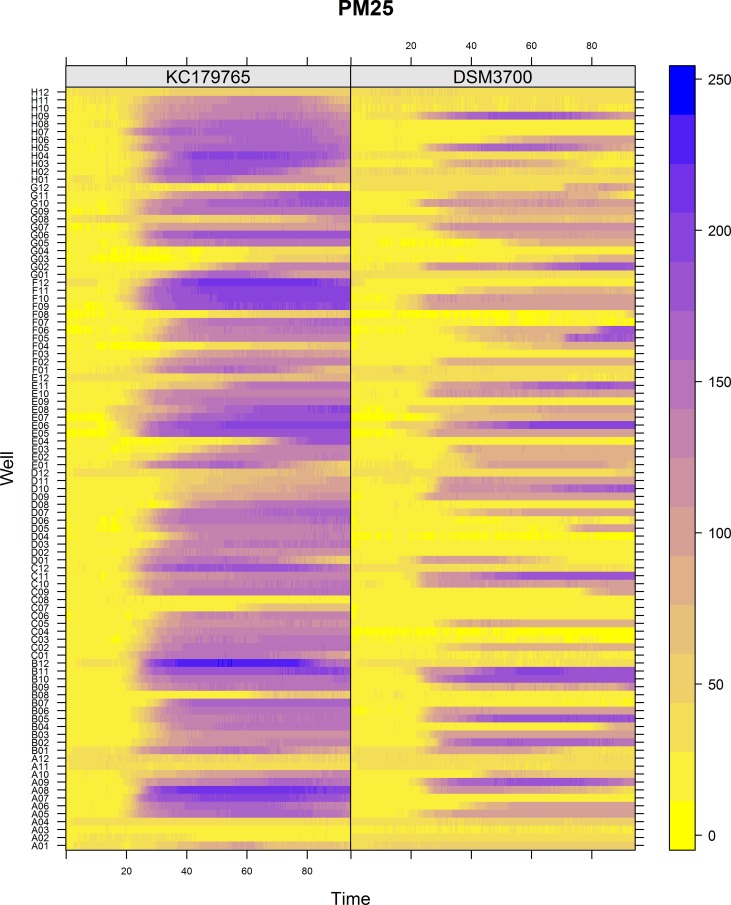
Visualization of *N*. *fischeri* DSM3700 and *N*. *fischeri* KC179765 growth kinetics on plate PM25. The growth values are represented by a color range as given in the scale bar on the right of the figure. The growth value increases from the yellow to the blue color. Yellow color represents growth inhibition.

The Biolog PM chemical sensitivity panels for fungi (PMs 21–25) contain 120 assays of chemical sensitivity. Each chemical sensitivity assays was used at four increasing doses of the tested chemical. The results of performing kinetic analysis are presented at Figs 1, 2, 3, 4 and 5. The chemical sensitivity profile analysis showed diversity between compared isolates. However, the results indicated that in general the growth inhibition of tested isolates increased with the increasing doses of chemical agents. The results indicated that both isolates overcame influence of chemical agents during first 24-incubation hours. The most surprising finding in our results was that fresh isolate of *N*. *fischeri* KC179765 had completely different and expanded chemical sensitivity profile when compared to long-stored isolate (*N*. *fischeri* DSM3700). As our results indicate, this isolate has a higher capacity to overcome almost all of the tested chemical agents. This was found to be particularly pronounced in the case of lower doses of chemicals. When an isolate achieves such increased capacity to utilize a wide spectrum of chemical agents, it is more likely to colonize plants, fruit or agricultural crops and this might explain why this isolate can be resistant to fungicides. Although the phenomenon of antifungal resistance is still of major concern in agriculture, according to Vandeputte *et al*. [18] antifungal agents resistance appears to be result of point mutations in either chemical compounds targets or transcription factors regulating the mechanisms of resistance. This could explain the lower chemical resistance of long-stored isolate of *N*. *fischeri* (DSM3700) in comparison of fresh-isolated one (KC179765). Fresh isolate was exposed to a wide range of chemicals, fungicides and competition with other microorganisms in the environment. The lack of exposure to such factors could have an impact on possible absence of particular chemical resistance mechanisms or on losing the resistance genes in long-stored isolate. Therefore, this isolate was very sensitive even for the lowest doses of chemical agents, what is presented at Figs 1, 2, 3, 4 and 5.

**Fig 6:**
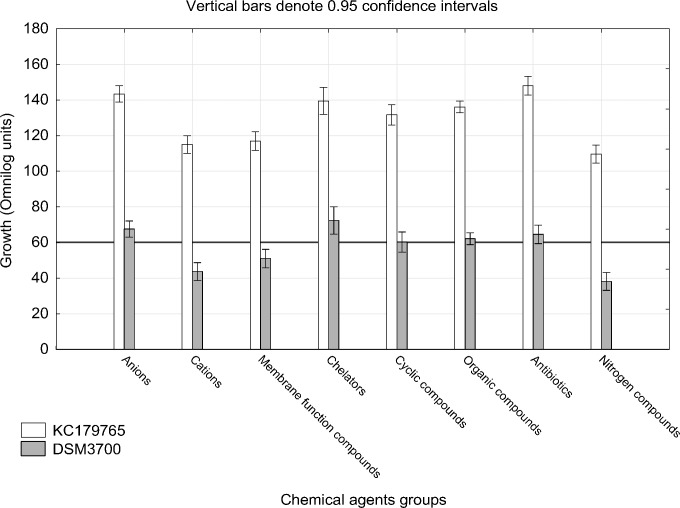
Visualization of *N*. *fischeri* DSM3700 and *N*. *fischeri* KC179765 growth in occurrence of each studied groups of chemical compounds. The scale represents growth values (Omnilog units) after 72h of incubation. The horizontal line at value of 60 represents the growth threshold considered as positive.

**Fig 7:**
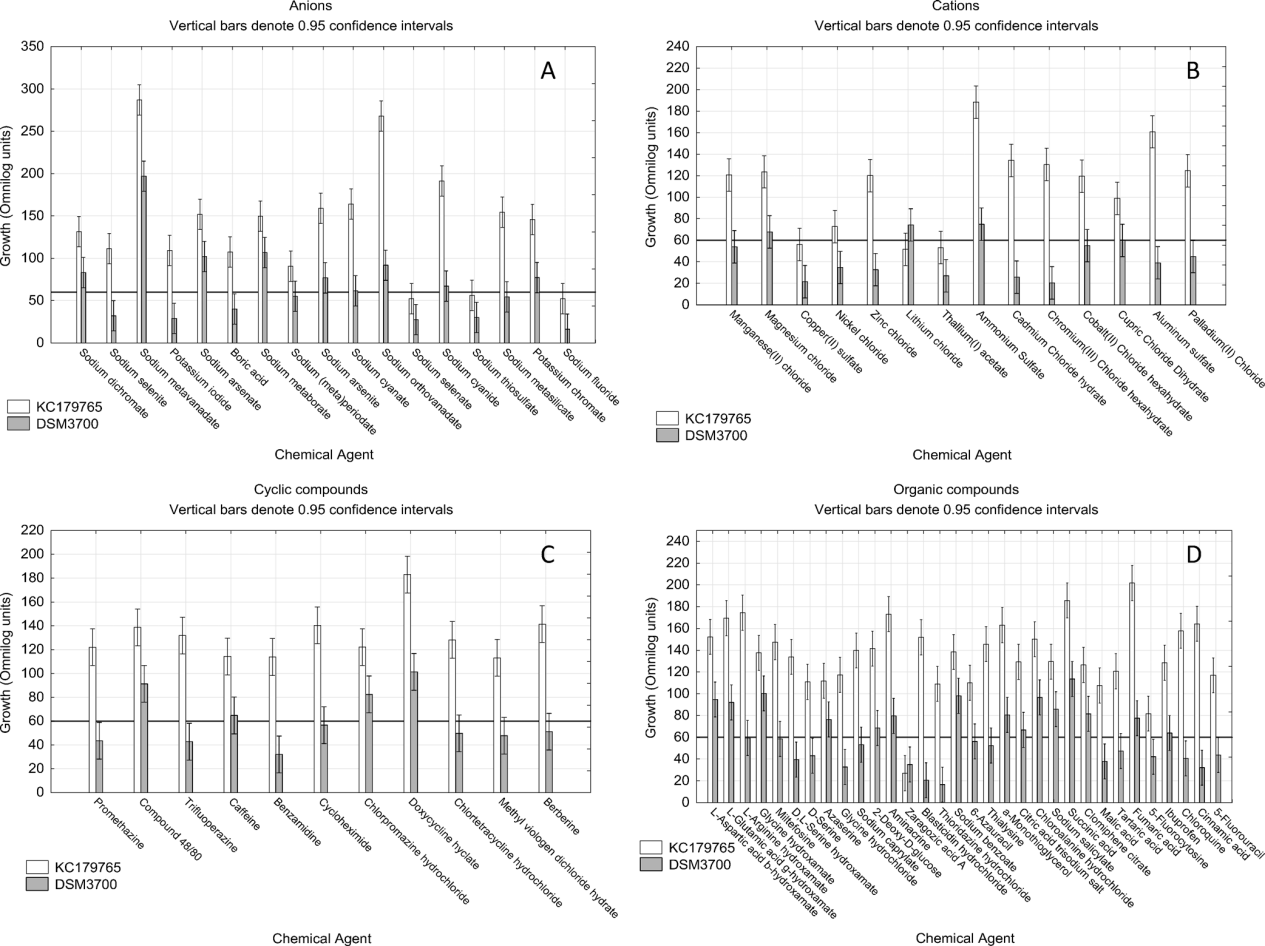
Visualization of *N*. *fischeri* DSM3700 and *N*. *fischeri* KC179765 growth in occurrence of toxic anions, cations, cyclic and organic compounds. The scale represents growth values (Omnilog units) after 72h of incubation. The horizontal line at value of 60 represents the growth threshold considered as positive.

The results of growth intensity of tested isolates for particular chemicals groups after 72 incubation hours are presented at Fig 6. We have observed, that *N*. *fischeri* KC179765 was more resistant to each tested group of chemicals. We found that three out of eight studied chemical groups were the most efficient in growth inhibition for both isolates. These groups are nitrogen compounds, toxic cations and membrane function compounds. We have observed that chelators and toxic anions have the lowest inhibitory effect. The inhibitory effect of studied antibiotics for *N*. *fischeri* KC179765 seem to be much lower than for *N*. *fischeri* DSM3700, when compared to other groups. Overall, the antibiotics, chelators and anionic compounds should be avoided in any interventions designed to control *N*. *fischeri*, as these group do not affect this species. However, the effects of anionic compounds should be analyzed separately for each substances due to very strong inhibiting influence of sodium selenate, sodium thiosulfate and sodium fluoride against both tested isolates (Fig 7A).

### 3.1. Toxic anions, cations, cyclic and organic compounds

Out of 17 of examined toxic anions, we found that 8 of them stopped the growth at least one or both of studied isolates (Fig 7A). Sodium selenite, potassium iodide, boric acid, sodium (meta)periodate and sodium metasilicate had the inhibitory effect only on *N*. *fischeri* DSM3700, while sodium selenate, sodium thiosulfate and sodium fluoride had this effect on both isolates. Apart of those three compounds, fresh isolate *N*. *fischeri* KC179765 was found to be more resistant to the remaining studied substances, than long-stored isolate *N*. *fischeri* DSM3700.

We have analyzed the effect of 14 toxic cations. Results allowed us to find that 12 of them had the strong inhibitory effect on either studied isolates (Fig 7B). Manganese(II) chloride, nickel chloride, zinc chloride, cadmium chloride hydrate, chromium(III) chloride hexahydrate, cobalt(II) chloride hexahydrate, cupric chloride dihydrate, aluminum sulfate and palladium(II) chloride affected the growth of long-stored isolate *N*. *fischeri* DSM3700. Lithium chloride effectively inhibited only the fresh isolate *N*. *fischeri* KC179765, while copper(II) sulfate and thallium(I) acetate had stopped both isolates. Apart of the last two mentioned substances, the inhibitory effect of other cationic substances on *N*. *fischeri* DSM3700 and *N*. *fischeri* KC179765 was very strong and moderate, respectively.

Fresh isolate of *N*. *fischeri* KC179765 appeared to be resistant to every one of 11 studied cyclic compounds. On the contrary, long-stored isolate *N*. *fischeri* DSM3700 was very vulnerable to appearance of this group of compounds. 7 of them: promethazine, trifluoperazine, benzamidine, cycloheximide, chlortetracycline hydrochloride, methyl viologen dichloride hydrate and berberine had very strong inhibitory effect (Fig 7C).

The studied organic compounds was the largest group of chemicals agents. It consisted of 32 chemical compounds of very differentiated effect on studied isolates growth. We have found one substance, zaragozic acid A, that was able to inhibit both studied isolates and 15 substances that showed strong inhibition on *N*. *fischeri* DSM3700 (Fig 7D). These substances were L-arginine hydroxamate, miltefosine, D,L—serine hydroxamate, D- serine, glycine hydrochloride, sodium caprylate, blasticidin hydrochloride, thioridazine hydrochloride, 6-azuracil, thialysine, malic acid, tartaric acid, 5-fluorocytosine, chloroquine, cinnamic acid and 5-fluorouracil.

### 3.2. Membrane function, chelators, antibiotics and nitrogen compounds

**Fig 8:**
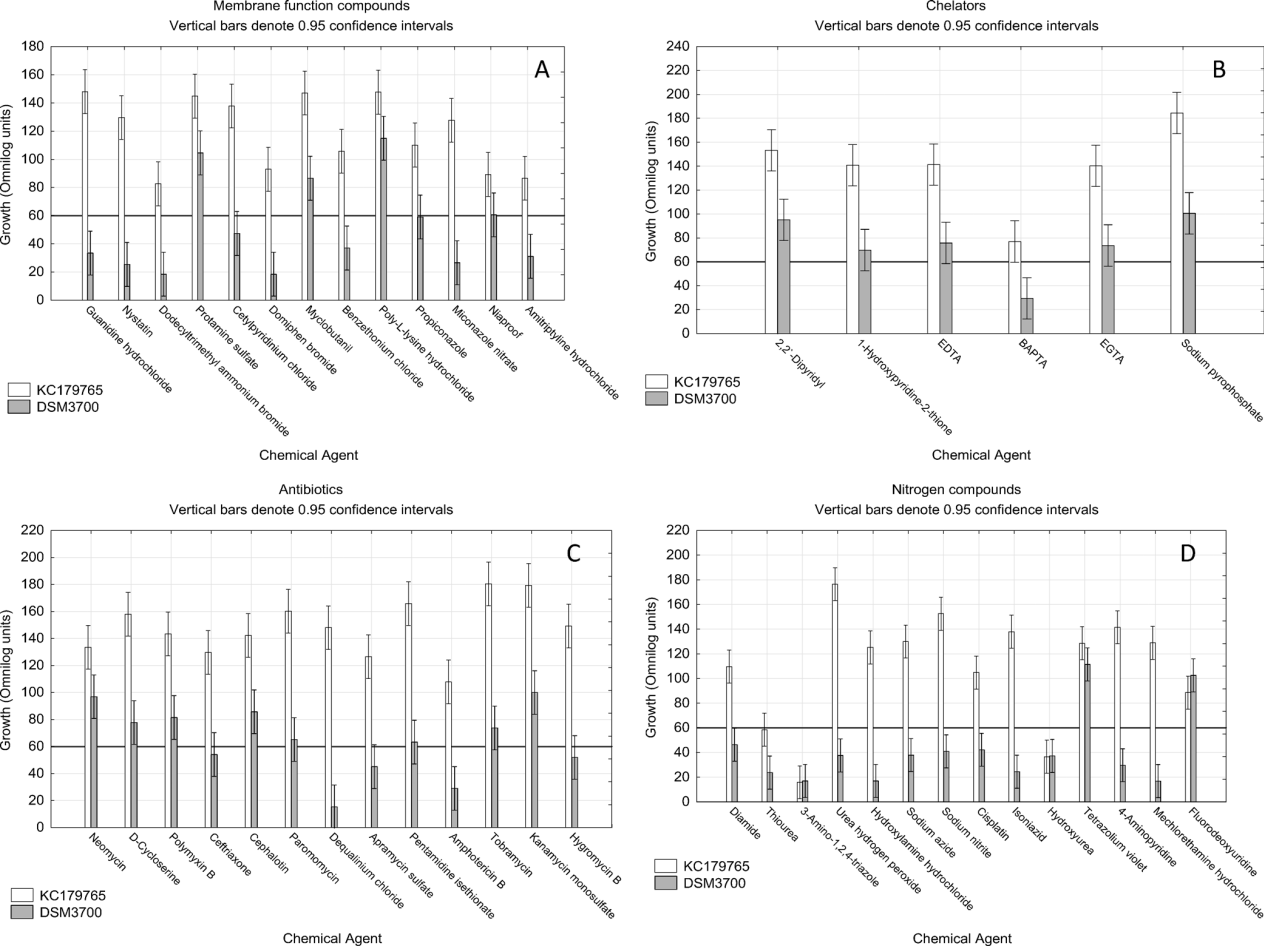
Visualization of *N*. *fischeri* DSM3700 and *N*. *fischeri* KC179765 growth in occurrence of membrane function, chelators, antibiotics and nitrogen compounds. The scale represents growth values (Omnilog units) after 72h of incubation. The horizontal line at value of 60 represents the growth threshold considered as positive.

The obtained results show very intensive inhibitory effect of membrane function compounds on long-stored isolate *N*. *fischeri* DSM3700 (Fig 8A). Out of 13 studied substances, only 4 were not able to stop the growth of this isolate. However, none of the studied substances had inhibiting impact on fresh *N*. *fischeri* KC179765 growth strong enough to consider it as the stopping of growth.

We observed, that fresh *N*. *fischeri* KC179765 was generally more resistant to chelators than long-stored *N*. *fischeri* DSM3700 isolate. The only one substance that had the inhibitory effect on either of isolates was 1,2-bis(o-aminophenoxy)ethane-N,N,N',N'-tetraacetic acid (BAPTA), which had stopped the growth of *N*. *fischeri* DSM3700 (Fig 8B).

We studied the impact of 13 antibiotics on growth of two *N*. *fischeri* isolates. We have found that fresh isolate *N*. *fischeri* KC179765 has developed strong resistance to all of studied antibiotics compounds (Fig 8C). Long-stored isolate *N*. *fischeri* DSM3700 was strongly inhibited by 5 antibiotics: ceftriaxone, dequalinium chloride, apramycin sulfate, amphotericin B and hygromycin B.

The group of nitrogen compounds appeared to be the most inhibiting to both isolates. Out of 14 studied compounds, 3 successfully stopped the growth of both isolates. These compounds were thiourea, 3-amino-1,2,4-triazole and hydroxyurea. We found *N*. *fischeri* DSM3700 to be more resistant than *N*. *fischeri* KC179765 to fluorodeoxyuridine, however growth of both isolates in these wells was considered to be positive (Fig 8D).

### 3.3. Area of growth inhibition

**Fig 9:**
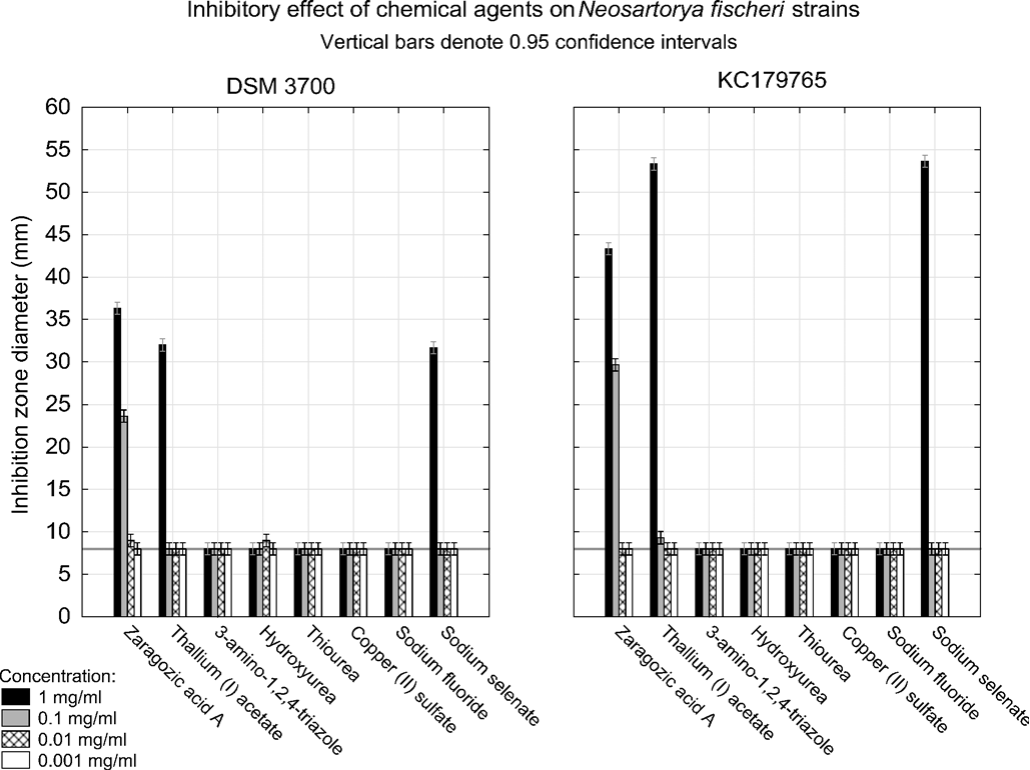
Visualization of inhibitory effect of zaragozic acid A, thallium(I) acetate, 3-amino-1,2,4-triazole, hydroxyurea, thiourea, copper(II) sulfate, sodium fluoride and sodium selenate on *N*. *fischeri* DSM3700 and *N*. *fischeri* KC179765 with hole-plate zone of growth inhibition method. The scale represents inhibition zone diameter (millimeters) after 72h of incubation. The horizontal line at value of 8 represents the hole diameter in the medium and is considered as threshold in growth inhibition.

Out of 8 studied substances, we found that only three of them presented inhibitory effect on tested isolates (Fig 9). Zaragozic acid A in concentration of 1mg/ml caused inhibition of both isolates growth expressed as 36.67 and 43.34 mm inhibition zones for *N*. *fischeri* DSM3700 and *N*. *fischeri* KC179765, respectively. The inhibition was present also in concentration of 0.1mg/ml which was expressed as 23.67 and 29.67 mm of growth inhibition zones for *N*. *fischeri* DSM3700 and *N*. *fischeri* KC179765, respectively. Lower concentrations of the compound had no inhibitory effect on both tested isolates. Thallium(I) acetate in concentration of 1mg/ml resulted in 32.00 and 53.34 mm of fungal growth inhibition zones, while lower concentrations had no effect on growth of both *N*. *fischeri* isolates. Sodium selenate in concentration of 1mg/ml caused 31.67 and 53.67 mm of growth inhibition zones for both tested isolates. The other studied substances: 3-amino-1,2,4-triazole, hydroxyurea, thiourea, copper(II) sulfate and sodium fluoride had no inhibitory effect on *N*. *fischeri* in any of tested concentrations.

## Discussion

Thermal death rates of *N*. *fischeri* ascospores under the influence of organic acids and preservatives were described in literature. It is known that citric and tartaric acids exhibit the destruction of ascospores in fruit juices. The preservatives like potassium sorbate and sodium benzoate are also used to the control of this fungus in fruit juices [19,20]. Delgado *et al*. [21] reported that hydrogen peroxide must be considered to reduce the probability of packages contamination by *N*. *fischeri*. There are no papers concerning antibiotics and fungicides influence on growth reduction of this fungus, but there are a lot of information that *N*. *fischeri* can be used as producer of antibiotics [22,23] and antifungal proteins [24–26]. In recent years the occurrence of fungal infections has been increasing everywhere, which may be explained by changing climatic conditions and resistance of fungi to fungicides due to their extensive use in agriculture [27,28]. Bromley *et al*. [28] reported that agricultural use of the most dominant class of antifungal agents–azoles, may lead to resistance in environmental fungi of clinical importance. They isolated also azole-resistant of *N*. *fischeri* species. In these cases it is reasonable to carry out study that can lead to the control of *N*. *fischeri* by finding substances that can be used as active compounds of chemical agents, including fungicides.

The presented results suggest that chemical sensitivity phenomics of newly isolated fresh *N*. *fischeri* isolate differs significantly from long-stored isolate. 62 out of 120 studied substances effectively inhibited growth of long-stored isolate *N*. *fischeri* DSM3700 whereas they did not have influence on growth of fresh environmental *Neosartorya fischeri* isolate (KC179765).

According to studies with Biolog PM Platform (Figs 7 and 8) the substances able to prevent both studied isolates from growth (*N*. *fischeri* KC179765 and *N*. *fischeri* DSM3700) were sodium selenate, sodium thiosulfate, sodium fluoride, copper(II) sulphate, thallium(I) acetate, zaragozic acid A, thiourea, 3-amino-1,2,4- triazole and hydroxyurea. However, results of further verifying studies involving hole-plate zone of inhibition method on PDA medium showed that only zaragozic acid A in concentrations of 1 mg/ml and 0.1 mg/ml and thallium(I) acetate and sodium selenate in concentration of 1.mg/ml had inhibitory effect on tested isolates growth.

Zaragozic acid A, sodium fluoride, 3-amino-1,2,4- triazole, copper(II) sulphate, and thiourea are known for their biocide properties [29–37], although their impact on *N*. *fischeri* has not been studied yet. According to our study, zaragozic acid A suppresses *N*. *fischeri* growth.

The inhibitory effect of zaragozic acid A on *N*. *fischeri* may be related to its strong inhibitory effect on the squalene synthase which also affects the sterol synthesis in organisms [29,38]. Sterols are important membrane components and precursors for the synthesis of powerful bioactive molecules. Sterol biosynthesis is a crucial pathway in eukaryotes leading to the production of ergosterol in fungi and is required for heat resistance [39]. Therefore, fungicides containing zaragozic acid A could be tested as specific and dedicated for heat resistant fungi elimination. We hypothesize that the fungicides with chemical agent mentioned above as active compound could be used in antifungal protection against heat resistant *N*. *fischeri* species.

Sodium selenate appeared to be good *N*. *fischeri* growth inhibitor. Selenium compounds are considered as safe for human health, however in concentrations over 2.4 milligrams of selenium per day it is considered as toxic [40,41]. Sodium selenate is the active ingredient often used in shampoos, and known as an antifungal agent in fungal infection as well as a cytostatic agent, slowing the growth hyperproliferative cells. There are no studies concerning heat resistant fungal growth inhibition by sodium selenate, but our results indicated its potential as active compound of fungicides against *N*. *fischeri* species. The growth inhibition of *N*. *fischeri* isolates caused by sodium selenate was confirmed by both PM and hole-plate methods. According to this study sodium selenate is a potent and promising *N*. *fischeri* oriented fungicide. Moreover, because selenium compounds like sodium selenate are used as fertilizers for selenium-poor soils [42] selenium-based fungicide could be safer for the environment than other toxic compounds.

In this study thallium(I) acetate showed promising inhibitory effects on *N*. *fischeri*. There is no studies concerning its use as a fungicide, however its toxic and genotoxic effect on human health [43,44] makes this substance very hazardous to use as a possible fungicide in food croplands.

Hydroxyurea, classified as ribonucleotide reductase M2, has not been yet reported in use as a fungicide. This study reports antifungal activity of hydroxyurea against *N*. *fischeri*. The inhibitory effect of hydroxyurea may be related to its ability to deter DNA synthesis [45]. Hydroxyurea is mutagenic in vitro to bacteria, fungi, protozoa, and mammalian cells [46]. Although because of possible negative impact on human health [47], hydroxyurea is used as an antineoplastic drug for treatment of HIV, cancer, and myeloproliferative diseases [48]. However, more studies concerning its antifungal activity and safety should be performed.

3-amino-1,2,4- triazole (3-AT), commonly known as amitrole, is a substance that usage is regulated by European Commission and can be used only as a herbicide [49]. Several derivatives of 1,2,4-triazoles have been reported to exhibit antifungal activity [50]. The inhibitory effect and impact on *N*. *fischeri* growth may be connected with 3-ATs reported effect on histidine biosynthesis, through competitive inhibition of imidazoleglycerol-phosphate dehydratase [51,52]. Moreover, it is reported to block the biosynthesis of riboflavin in plants [53]. 3-AT is also a potent catalase inhibitor [54].

Thiourea has been reported for its genotoxic properties on yeast [55,56]. However the studies concerning its effect on human health collected in World Health Organization [57], show that there is no proof of negative effect of thiourea on human health. In addition, some thiourea derivatives are known to be associated with a wide range of biological activities such as analgesic, antitumor, antioxidant, anticonvulsant, and anti-HIV properties [58–66]. However, antibacterial and antifungal activities of thiourea derivatives have been less widely documented [67–69]. Our results based on PM microplates suggested that thiourea could be used as potential antifungal agent in heat resistant *N*. *fischeri* species elimination, however this effect has not been confirmed by hole-plate method.

Copper(II) sulphate is well known [36] and allowed to use by European Commission as fungicide [49]. Its inhibitory effect on *N*. *fischeri* may be based on toxic properties of Cu^2+^ ions [70]. It was reported that fungi may adapt to the occurrence of copper in environment [71].

The fungicide effect of sodium fluoride is known [72]. However, some fungi had developed mechanism allowing them to survive in high fluoride concentrations. This mechanism involves the expression of *FEX* gene and synthesis of FEX protein [73]. In order to use fluoride compounds as fungicide, genetic studies directed on occurrence of FEX gene in fungi should be performed. Latest study reported that inhibitory effect of sodium fluoride was found against fungus *Fusarium oxysporum* [74]. The results from our PMs study demonstrated that this substance could also have antifungal activity to the heat resistant *N*. *fischeri* fungi, however this effect has not been confirmed using hole-plate method. Therefore based on this results we suppose that the influence of sodium fluoride on heat resistant fungi growth is depended on the availability of nutrients in the environment. In rich-nutrients environment there is no growth inhibition (PDB medium), while in poor-nutrients environment the inhibitory effect is present (PM plates medium).

The provided experimentations are complex evaluation and screening of the in vitro effect of different chemicals on growth of *Neosartorya fischeri*. Results of performing PM assay on *N*. *fischeri* provide important information on phenomics of this important heat resistant fungus. The hole-plate zone of inhibition analysis allows to verify data obtained with PMs assay and to determine the effective concentrations of chemical agents. The presented study based on both PMs and hole-plate methods showed that the use of sodium selenate, thallium(I) acetate and zaragozic acid A could be considered as a potential fungicides against heat resistant *N*. *fischeri*. For the control of the heat resistant fungi, these compounds could potentially be used alone or in combination with other safe treatments. However, these compounds are not considered as GRAS (Generally Recognized As Safe) by the FDA (American Food and Drug Administration). Therefore, there is a need to evaluate the effect of these compounds on seeds and plants. These results provided information of the most promising antifungal agents against *N*. *fischeri*. Received data can be used for detailed comparison of other isolates and species of heat resistant fungi in further studies.

This study also presents results on comparison of Biolog PMs chemical sensitivity assay with traditional hole-plate zone inhibition method. Results did not perfectly match those achieved with Biolog assay. Confirmation of PMs Biolog results by traditional hole-plate method were observed with regard to three above mentioned potential fungicides compounds. We hypothesize that it could be caused by usage of media poorer in nutrients in Biolog assay, or by differences in chemical agents concentration, as exact concentrations in Biolog assay are unpublished, therefore unknown. We suppose that fungi grown in optimal nutrient conditions tend to be more chemical resistant. The Biolog PMs microplate was effective and time saving alternative method for determining *N*. *fischeri* resistance/sensitivity to chemicals. Because quantitative assays were less labor intensive and faster in PMs microplates than in traditional method this approach can be very useful in screening study of chemical sensitivity of heat resistant fungi. Then the most promising chemical agents influence on fungal growth should be confirmed by conventional hole-plate methods.

In summary, our results present the most comprehensive analysis of chemical sensitivities of heat-resistant *N*. *fischeri* isolates. These findings could be used for designing better prevention and intervention methods against *N*. *fischeri* and also for formulating new active compounds of specific fungicides dedicated to the control of this species.

## Supporting Information

S1 Table Chemical content of each well in PM chemical sensitivity panel. (PDF)

S2 Table Studied chemical compound divided into structure and functional groups. (PDF)
