# Supplementary material for: Comparison of Chemical Sensitivity of Fresh and Long-Stored Heat Resistant Neosartorya fischeri Environmental Isolates Using BIOLOG Phenotype MicroArray System
Source: PLoS One. 2016 Jan 27;11(1):e0147605. doi: 10.1371/journal.pone.0147605 (PMC4729462; doi:10.1371/journal.pone.0147605)
Supplement: S1 Table — (PDF) [file pone.0147605.s001.pdf]

S1 Table. Chemical content of each well in PM chemical sensitivity panel.

	Plate				
Well	PM21	PM22	PM23	PM24	PM25
A 14	Guanidine hydrochloride	L-Glutamic acid ghydroxamate	Benzethonium chloride	Apramycin sulfate	Hydroxyurea
A 58	2,2`-Dipyridyl	Sodium metavanadate	Chlorpromazine hydrochloride	Aminacrine	Tobramycin
A 912	Promethazine	Caffeine	Ammonium sulfate	Zaragozic acid A	Niaproof
B 14	Nystatin	L-Arginine hydroxamate	Cadmium chloride hydrate	Blasticidin hydrochloride	Chloroalanine hydrochloride
B 58	Dodecyltrimethyl ammonium bromide	Glycine hydroxamate	Dequalinium chloride	Thioridazine hydrochloride	Tetrazolium Violet
B 912	Protamine sulfate	Potassium iodide	Doxycycline hyclate	Sodium Benzoate	Kanamycin monosulfate
C 14	Cetylpyridinium chloride	3-Amino-1,2,4- triazole	Glycine hydrochloride	Chlortetracycline hydrochloride	4-Aminopyridine
C 58	Domiphen bromide	Miltefosine	Hydroxylamine hydrochloride	Sodium metasilicate	Amitriptyline hydrochloride
C 912	L-Aspartic acid β-hydroxamate	D,L-Serine hydroxamate	Poly-L-lysine hydrochloride	Pentamidine isethionate	Citric acid
D 14	1-Hydroxypyridine2-thione	Polymyxin B	Chromium(III) chloride hexahydrate	6-Azauracil	Mechlorethamine hydrochloride
D 58	EDTA	Urea hydrogen peroxide	Cobalt(II) chloride hexahydrate	Potassium chromate	Hygromycin B
D 912	Sodium dichromate	Sodium Arsenate	Copper(II) chloride dihydrate	Thialysine	Fluorodeoxyuridine
E 14	Compound 48/80	Ceftriaxone	Sodium metaborate tetrahydrate	α-Monothioglycerol	Sodium salicylate
E 58	Manganese(II) chloride	BAPTA	Sodium (meta)periodate	EGTA	Succinic acid
E 912	Magnesium chloride	D-Serine	Sodium arsenite	Sodium pyrophosphate decahydrate	Clomiphene citrate
F 14	Copper(II) sulfate	Azaserine	Sodium azide	Propiconazole	Malic acid

F 58	Neomycin	Lithium chloride	Sodium caprylate	Methyl viologen dichloride hydrate	Tartaric acid
F 912	D-Cycloserine	Boric acid	Sodium cyanate	Sodium fluoride	Fumaric acid
G 14	Sodium Selenite	Benzamidine	Sodium Nitrite	Cisplatin	5-Fluorocytosine
G 58	Nickel chloride	Cycloheximide	Sodium orthovanadate	Aluminum sulfate	Palladium(II) chloride
G 912	Trifluoperazine	Thallium(I) acetate	2-Deoxy-Dglucose	Berberine chloride	Ibuprofen
H 14	Diamide	Cephalotin	Sodium selenate	Isoniazid	Chloroquine
H 58	Thiourea	Paromomycin	Sodium cyanide	Amphotericin B	Cinnamic acid
H 912	Zinc chloride	Myclobutanil	Sodium thiosulfate	Miconazole nitrate	5-Fluorouracil
