# Supplementary material for: Comparison of Chemical Sensitivity of Fresh and Long-Stored Heat Resistant Neosartorya fischeri Environmental Isolates Using BIOLOG Phenotype MicroArray System
Source: PLoS One. 2016 Jan 27;11(1):e0147605. doi: 10.1371/journal.pone.0147605 (PMC4729462; doi:10.1371/journal.pone.0147605)
Supplement: S2 Table — (PDF) [file pone.0147605.s002.pdf]

S2 Table. Studied chemical compound divided into structure and functional groups.

Group	Substance				
Anions	Sodium dichromate	Sodium Selenite	Sodium metavanadate	Potassium iodide	Sodium arsenate
	Sodium metaborate	Sodium (meta)periodate	Sodium Arsenite	Sodium Cyanate	Sodium Orthovanadate
	Sodium Cyanide	Sodium Thiosulfate	Sodium metasilicate	Potassium Chromate	Sodium Fluoride
	Boric acid	Sodium Selenate			
Cations	Dodecyltrimethyl ammonium bromide	Manganese(II) chloride	Magnesium chloride	Copper(II) sulfate	Nickel chloride
	Lithium chloride	Thallium(I) acetate	Ammonium Sulfate	Cadmium Chloride hydrate	Chromium(III) Chloride hexahydrate
	Cupric Chloride Dihydrate	Aluminum sulfate	Palladium(II) Chloride	Zinc chloride	Cobalt(II) Chloride hexahydrate
Membrane function compounds	Guanidine hydrochloride	Nystatin	Dodecyltrimethyl ammonium bromide	Protamine sulfate	Cetylpyridinium chloride
	Myclobutanil	Benzethonium Chloride	Poly-L-lysine hydrochloride	Propiconazole	Miconazole Nitrate
	Amitriptyline hydrochloride	Domiphen bromide	Niaproof		
Chelators	2,2`-Dipyridyl	1-Hydroxypyridine2-thione	EDTA	BAPTA	EGTA
	Sodium Pyrophosphate Decahydrate				
Cyclic compounds	Promethazine	Compound 48/80	Caffeine	Benzamidine	Cycloheximide
	Doxycycline Hyclate	Chlortetracycline hydrochloride	Methyl Viologen Dichloride hydrate	Berberine chloride	Chlorpromazine hydrochloride
Organic compounds	L-Aspartic acid bhydroxamate	L-Glutamic acid ghydroxamate	L-Arginine hydroxamate	Glycine hydroxamate	Miltefosine
	D-Serine	Azaserine	Glycine hydrochloride	Sodium Caprylate	2-Deoxy-Dglucose
	Zaragozic acid A	Blasticidin hydrochloride	Thioridazine hydrochloride	Sodium Benzoate	6-Azauracil
	α-Monothioglycerol	Citric acid Trisodium Salt	Chloroalanine hydrochloride	Sodium salicylate	Succinic acid

	Malic acid	Tartaric acid	Fumaric acid	5-Fluorocytosine	Ibuprofen
	Cinnamic acid	5-Fluorouracil	D,L-Serine hydroxamate	Aminacrine	Thialysine
	Clomiphene citrate	Chloroquine			
Antibiotics	Neomycin	D-Cycloserine	Polymyxin B	Ceftriaxone	Cephalotin
	Dequalinium Chloride	Apramycin Sulfate	Pentamidine Isethionate	Amphotericin B	Tobramycin
	Hygromycin B	Paromomycin	Kanamycin Monosulfate		
Nitrogen compounds	Diamide	Thiourea	3-Amino-1,2,4triazole	Urea hydrogen peroxide	Hydroxylamine hydrochloride
	Sodium Nitrite	Cisplatin	Isoniazid	Hydroxyurea	Tetrazolium Violet
	Mechlorethamine hydrochloride	Fluorodeoxyuridine	Sodium Azide	4-Aminopyridine	
